# Supplementary material for: Investigating Potential Cancer Therapeutics: Insight into Histone Deacetylases (HDACs) Inhibitions
Source: Pharmaceuticals (Basel). 2024 Mar 29;17(4):444. doi: 10.3390/ph17040444 (PMC11054547; doi:10.3390/ph17040444)
Supplement: Supplementary file 1 [file pharmaceuticals-17-00444-s001.zip › pharmaceuticals-2861525-supplementary.pdf]

Supplementary data

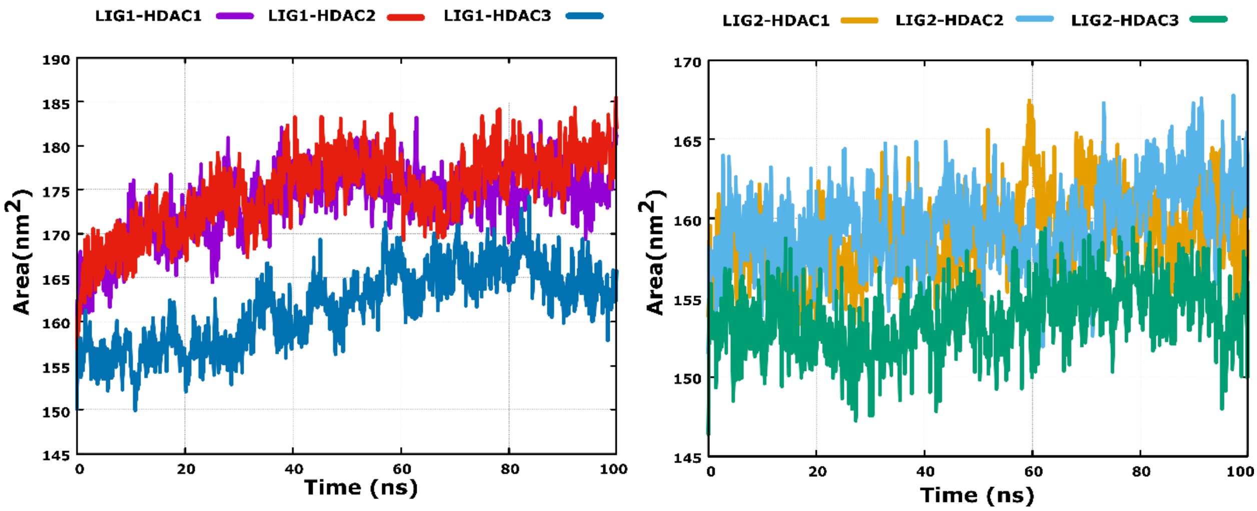

Figure S1. Solvent accessible surface area (SASA) of the HDAC enzymes associated with LIG1 and LIG2 for 100 ns of MD simulation.
